# 

**Published:** 1890-05

**Authors:** 


### AMERICAN JOURNAL

—OF—

### DENTAL SCIENCE,

—EDITED BY—

F. J. S. GORGAS, M. D., D. D. S.

VOLUME XXIV—Third Series.

1890-91.

SURGEON GENL'S OFFICE
141137
LIBRARY

BALTIMORE:
SNOWDEN & COWMAN,

LONDON:
TRUBNER & CO. 60 PATERNOSTER ROW.

1891.

### Index to Volume XXIV. Third Series.

A Study of Electricity, with the View of Comprehending its Application in Dentistry, - - - 31
A Case in Practice, - - - - - 48
Aseptic and Antiseptic Dentistry, - - - 81
A Dental Operation in which Teeth Buried in the Jaw are Removed, - - - - - 95
American Dental Association, - - - 140
Acromegaly, - - - - - - 141
A Cheshire Farmer and his False Teeth, - - 143
American Academy of Dental Science, - - - 217
American Dental Association, - - - 219
An Anomalous Case on Salivary Calculus, - - 279
A Practical Method of Electro Gilding Gold Dentures, &c, 288
Affections Likely to Occur in the Alveolar Process, - 322
A Local Anæsthetic Formula, - - - - 335
A Note Concerning the Action of Chloroform, - 369
A Note Concerning the Action of Chloroform, - - 385
Antiquity of Dentistry, - - - - 426
A Cause of Dental Caries, - - - - 426
Art and Dentistry, - - - - - 430
A Tooth Drawn by a Hen, - - - - 469
Antiquity of Artificial Teeth, - - - 469
A Remarkable Surgical Operation, - - - 470
An Electric Plant, - - - - - 471
A Ready Inhaler, - - - - - - 476
Administration of Morphine by the Nostrils, - 478
A New Mixture for Use in Local Anæsthesia, - - 479
A Review of Copper Amalgam, - - - 498
Adhesion of Soft Palate to Pharynx, Operation; Cure, - 507
Atkinson Resolutions.—St. Louis Dental Society, - 575
Breathing through the Mouth a Cause of Decay of the Teeth, - - - - - - 142
Broken Instruments in Root Canals, - - - 503
Baltimore College of Dental Surgery, - - 572

BIBLIOGRAPHICAL:
A New Medical Dictionary, - - - - 190
A New Dental Journal, - - - 191
A Change of Editors, - - - - 191
A Treatise on the Irregularity of the Teeth and their Correction, - - - - - 427
Catching's Compendium of Practical Dentistry, 192
Descriptive Anatomy of the Human Teeth, - 383
Harris' Dictionary, - - - - - 522
The Student's Manual and Hand-Book for the Laboratory, - - - - - 192
The Dental Laboratory, - - - - 384
Transactions of the American Dental Association, 384
The Physician's Visiting List for 1891, - - 429
The Medical Bulletin Visiting List or Physician's Call Record, - - - - - 429
Changes of the Oral Secretions, - - - 16
Chloride of Methyl Spray, - - - - 90
Chicago Dental Society, - - - - 91
Calcification, - - - - - - 155
Current Dental Literature, - - - - 273
Cholera Precautions Suggested, - - - 282
Cocaine Anæsthesia Obtained by Means of Cataphoresis, 336
Coffee as a Disinfectant, - - - - 473
Chloroform as an Antiseptic, - - - - 474
Cures for Neuralgia of the Head, - - - 476
Campho-phenique, - - - - - 477
Codein, - - - - - - - 479
Dentistry in 1892, - - - - - 144
Dental Education, - - - - - 145
Dental Education - - - - - 201
Dangers from Necrosed Teeth, - - - 275
Dangers of Hypnotism, - - - - - 285
Danger in Exercise, - - - - - 286
Dr. Koch's Consumptive Cure, - - - - 300
Deformity of Face Treated with Apparatus, - 320
Dangers of Salivation from Calomel and Acids, - - 336
Dental Prosthesis, - - - - - - 527
Dental Medicaments with Special Reference to Antiseptics, 562

Euphorine, - - - - - - 333

Gold and Tin in Saving Teeth, - - - 116

Germs in a Baby's Jaw, - - - - - 189

Getting Ahead of St. Paul, - - - - 283

Hypnotism in Dentistry, - - - - 177

How to Deal with Another Man's Patients, - 277

Hypodermic Injections of Ergot in Facial Neuralgia, - 187

How to Introduce a Gutta-Percha Filling, - - 337

Hæmorrhage after Tooth Extraction and the Immediate Treatment, - - - - - - 525

Invitation to the Tenth International Medical Congress, 46

Impressions and Impression-Taking, - - 97

Immediate Root Fillings, - - - - 174

Identification by the Teeth, - - - - 234

Isococaine, a New Anæsthetic, - - - - 480

Illinois State Dental Society, - - - 520

Listerine, - - - - - - 240

Later Opinions on the Value of Koch's Discovery, - 431

Monthly Meeting of the American Academy of Dental Science, held at the Boston Medical Library Association Rooms, February 5, 1890, - - - 1

Missouri State Dental Association, - - - 41

Meeting of the American Dental Association, - - 42

Mouth-Breathing and the Teeth, - - - 47

Missouri State Dental Association, - - - 179

Mysteries of the Nerves, - - - - 238

Missouri Dental College, - - - - 516

Missouri Dental College Alumni, - - - 519

Missouri State Dental Association, - - - 570

Minnesota State Dental Association, - - 576

Notes on a Case of Syphilitic Cleft Palate, with its Treatment, - - - - - - 125

National Association of Dental Examiners, - 140

National Association of Dental Examiners, - - 182

National Association of Dental Faculties, - - 186

Normal Weight of Man, - - - - 335

Our Position at the Chair, - - - - 95

Observations on the Structure and Development of Ovarian Teeth, - - - - - 107

On the Dangers Arising from Syphilis in the Practice of Dentistry, - - - - - 241

On the Dangers Arising from Syphilis in the Practice of Dentistry, - - - - - - - 289

Olden-Time Artificial Teeth, - - - 447

Prosthetic Hygiene, - - - - - - 63

Post Graduate Dental Association of the United States, 89

Pyorrhœa Alveolaris Complicated with Necrosis, - 286

Primary Anæsthesia in Minor Operations, - - 331

Practical Suggestions, - - - - 471

Physiological Action of Thialdine, - - - 475

Pyorrhœa Alveolaris, - - - - 481

Root Filling in its Relation to Disease, - - 128

Resuscitation of the Apparently Drowned, - - 379

Retaining Points, - - - - - 432

Some Practical Points Learned at Society Meetings and Clinics, - - - - - - - 23

St. Louis Dental Society, - - - - 43

Some thoughts on Crown and Bridge-Work, - 193

Some Historic Points and Practical Hints on Crowning, 265

Stomatitis.—Diarrhœa in Infants, - - - 362

Some Practical Points Involved in the Relation of the Upper to the Lower Teeth, - - - - 455

Simple Method for Controlling Epistaxis, - - 465

Some Practical Suggestions about Plaster of Paris, - 510

The Dental Protective Association, - - 44

The Odontological Society of Great Britain, - 49

The Physiology and Treatment of Sensitive Dentine, - 73

The Law of the State of Virginia Regulating the Practice of Dentistry, - - - - 92

The Dental Mirror, - - - - - 96

The Care of the Vulcanizer, - - - 121

Tin and Gold in Combination for Filling Teeth, - 162

Tannic Acid. Its Internal Administration for Hemorrhage after Tooth Extraction, - - - 167

The First Permanent Molar, - - - - - 171

The Conservative Treatment of the Dental Pulp, - 208

The New Theory of the Action of Chloroform, - 212

The Dental Protective Association, - - 217

The Hypnotism Craze and its Dangers, - - 250

The Odontoblasts: Their Formation, Function and Power of Appropriation, - - - 252

The Chemistry of Dental Caries, - - 260

Trade in Cast-off Teeth, - - - 284

The Preparation and Filling of Roots at One Sitting, 309

The California State Board of Dental Examiners, and its Influence on Dental Education, - 324

The Kansas City Academy of Medicine, - 327

The Baltimore Dentists and the Census, - - 327

Treatment of Abscesses, - - - 394

The Effects of Mercury on the Teeth, - 417

Tannic Acid: Its Internal Administration for Hæmorrhage after Tooth Extraction, - - 422

The Treatment of Proximate Cavities, - - 433

Treatment of the Third Stage of Alveolar Abscess, 437

The Treatment of Proximate Surfaces, - - 440

The Application of Massage in the Treatment of Dental Pathological Conditions, - 450

The Dangers Arising from Syphilis in the Practice of Dentistry, - - - - - 461

The Combination of Tin and Gold as a Filling Material, - - - - - 467

The Youngest Doctor, - - - - 470

The Malleability of Gold, - - - - 470

The Nature of Koch's Lymph, - - - 472

The Application of Massage in the Treatment of Dental Pathological Conditions, - - 492

The Dental Pulp, - - - - 515

Teeth in Diabetes, - - - - - 528

The Odontological Society of Great Britain, - 529

The Obtunding of Sensitive Dentine, - - 545

The Treatment of Dead Teeth, - - - 551

The Dental Pulp, - - - - - 567

The Florida State Dental Association, - - 575

The Physiological Action of Sulphaldehyde, - 576

University of Maryland, Dental Department, - 573

Washington's Tooth, - - - - 282

Wandering Rash on Children's Tongues, - 334

Wonders of Surgery, - - - - 381

### Communications.

### MISSOURI STATE DENTAL ASSOCIATION.

The Executive Committee of the Missouri State Denta Association announce that its twenty-sixth annual meeting will will be held at Pertle Springs, Warrensburg, Johnson County, Missouri, commencing Tuesday, July 8th, 1890. Nothing has been left undone, that was in the power of the committee, to make the meeting both attractive and instructive; the clinics will constitute a special feature of the programme.

To the older members of the Association, many of whom have not been in attendance of late years, we would say this meeting is intended to serve as a reunion, and you are especially urged to be present.

To those of the profession in the State who are not members of the Association we extend a cordial invitation to attend and unite with us in the good work. Every reputable dentist in the State owes it to himself, his patrons and his profession to attend and aid in building up his State Association. None are compelled to apply for membership in order to receive its benefits, so make your arrangements to be in attendance whether you join the Association or not. To our brother dentists in other States we would simply say: You need no assurance of a welcome from Missouri, her arms are always open to you, and to as many of you as may honor us with your presence, we will endeavor to make the visit both pleasant and profitable. Reduced Hotel rates have been assured and those desiring rooms reserved in advance will be accommodated by addressing Mr. J. H. Christopher, Pertle Springs, Warrensburg, Mo.

OFFICERS FOR 1889-90.

President, Henry Fisher, St. Louis; 1st Vice-President, J. D. Patterson, Kansas City; 2nd Vice-President, J. P. Gray, Sedalia; Recording Secretary, John G. Harper, St. Louis; Corresponding Secretary, Wm. Conrad, St. Louis; Treasurer, James A. Price, Weston.

Executive Committee: J. F. McWilliams, W. L. Reed, W. H. Buckley.

Board of Censors: T. M. Nicholson, J. W. Whipple, J. F. McWilliams.

Committee on Ethics: W. H. Gaines, I. D. Pearce, C. V. Huff.

Publication Committee: H. S. Lowry, E. W. Stevens, W. E. Tucker.

Law: James A Price.

J. F. McWILLIAMS,
W. L. REED,
W. H. BUCKLEY,
*Ex. Com.*

WM. CONRAD, *Cor. Sec'y.*

---

### MEETING OF THE AMERICAN DENTAL ASSOCIATION.

The railroad arrangements are not all completed, but enough is known to assure at least the usual reduction of one and a third fare, on certificate plan, by all the different passenger associations.

Arrangements are being made for a special train from Chicago, which will leave here Sunday afternoon, and reach Excelsior Springs Monday morning. This will give the entire day, Monday, for the different sections to complete their reports.

All parties wishing to go in this special train will confer a favor by letting us know at once, so that we may know how many to arrange for. Just what rate will be secured for the round trip is not definitely settled, but we expect a low one.

A notice will be issued later giving exact time of starting and route selected.

Application has been made for reduced rates for the four associations, the American Dental Association, College Faculty Association, National Board of Dental Examiners and the Dental Protective Association. We are trying to get this rate good for ten days so that we need not adjourn before everything is finished.

Parties purchasing tickets should be sure to get receipt, showing that they have paid full fare going, this will enable them to get return ticket for one third regular fare.

J. N. CROUSE,
*Chairman Ex. Com.*
2231 Prairie Ave., Chicago.

---

EDITOR AMERICAN JOURNAL OF DENTAL SCIENCE,
BALTIMORE, MARYLAND.

*Dear Sir:*—At a meeting of the St. Louis Dental Society, held on Tuesday, May 20th, 1890, the following resolutions on the death of Dr. Homer Judd were adopted:

*Whereas*, The members of the St. Louis Dental Society have learned with great sorrow that death has removed from us our loved and honored associate, Dr. Homer Judd; and

*Whereas*, By reason of his great natural abilities, ripe scholarship, zeal, industry and integrity, he was recognized by the dental profession as one of its most influential members; a man who devoted his life to the honor and advancement of his profession; and

*Whereas*, During a long professional life, his relations with this Society have been such that it is our pleasure and duty to record our high appreciation of him; therefore

*Resolved*, That by the death of Dr. Homer Judd, the dental profession has been deprived of one of its most able and useful members, one whose influence for good will live forever.

*Resolved*, That we extend to his family our sincere sympathy in their great bereavement.

*Resolved*, That a copy of these resolutions be sent to the family of the deceased, and to the dental journals for publication.

W. H. EAMES,
HENRY FISHER,
A. J. PROSSER,
*Committee.*

MRS. E. E. CHASE, *Cor. Sec'y.*

---

### Editorial, Etc.

### THE DENTAL PROTECTIVE ASSOCIATION.

The next meeting of the National Association of Dental Examiners will be held in Excelsior Spring, Mo., on Monday evening, August 4th, at eight o'clock, and at other times during the week, between the sessions of the American Dental Association. It is important to have every State Board represented.

FRED. A. LEVY, D. D. S., *Sec'y*.

### INVITATION TO THE TENTH INTERNATIONAL MEDICAL CONGRESS.

[All communications must be directed to the General Secretary, Berlin NW., Karlstrasse 19.]

In accordance with the decision of the Ninth Congress at Washington, the Tenth International Medical Congress will be held at Berlin from the 4th to the 9th of August, 1890.

By the delegates of the German Medical Faculties and the chief Medical Societies of the German Empire, the undersigned have been appointed members of the General Committee of Organization. A Special Committee of Organization has also been appointed for each of the different sections, to arrange the scientific problems to be discussed at the meetings of the respective sections. An International Medical and Scientific Exhibition will also be held by the Congress.

We have the honor to inform you of the above decision, and at the same time cordially invite your attendance at the Congress. We should esteem it a favor if you would kindly extend this invitation to your friends in Medical Circles, as way may offer.

We beg to accompany our invitation by a copy of the Statutes and Programme, as also by the List of the intended sections and their Special Committees of Organization.

SECTION XVI.—There will be five general subjects for discussion:

1. Bromide of Ethyl Narcosis.
2. Pyorrhœa Alveolaris.
3. Micro Organisms in Caries.
4. Crown and Bridge Work.
5. Irregularities of Teeth.

Of these one has been assigned to Germany, one to England, one to France and two to America.

Dr. W. C. Barrett, of Buffalo, N. Y., has been selected by the General Committee to present the subject of Crown and Bridge Work. and E. S. Talbot that of Irregularities of Teeth. These five subjects, with the clinics and demonstrations by men appointed by the General Committee for that duty, will make up the official programme of the Section.

The other papers read will be selected from the voluntary contributions, and will be as many as time and the General Committee will permit.

The American officers of the Section are as follows: W. C. Barrett, M. D., D. D. S., Buffalo, N. Y.; J. Taft, M. D., D. D. S., Cincinnati, Ohio; C. S. Talbot, M. D., D. D. S., Chicago, Ill.; H. J. McKellops, D. D. S., St. Louis, Mo.

R. R. Andrews, D. D. S., of Cambridge, Mass., has been by the Board appointed Honorary Secretary.

A large number of American dentists has signified their intention to be present at the Congress.

DR. RUDOLF VIRCHOW,
*President.*

DR. VON BERGMANN,
" LEYDEN,
" WALDEYER,
*Vice-Presidents.*

DR. LASSAR,
*Secretary General.*

---

### Monthly Summary.

### To Readers and Correspondents.

Communications intended for publication, and exchange journals and papers, should be addressed to the Editor of The American Journal of Dental Science, No. 845 N. Eutaw St. Baltimore, Md. All letters relating to the business of the Journal, or containing cash remittances, to be directed to Messrs. Snowden & Cowman. No. 9 W. Fayette Street, Baltimore, Md. Articles for publication should be received by the Editor at least two weeks previous to the first of the month on which the number in which it is designed for them to appear, is issued.

### CONTENTS:

Article I.—Monthly meeting of the American Academy of Dental Science, held at the Boston Medical Library Associotion Rooms, February 5, 1890. - - 1

" II.—Changes of the Oral Secretions. By G. T. Thurer. 16

" III.—Some Practical points learned at Society Meetings and Clinics. By Dr. B. A. R. Ottolengui. - - 23

" IV.—A study of electricity, with the view of comprehending its application in Dentistry. By H. E. Roberts, D. D. S. - - - - - 31

COMMUNICATIONS.

Missouri State Dental Association. - - - - - 41

Meeting of the American Dental Association. - - - 42

St. Louis Dental Society. - - - - - - - 43

EDITORIAL.

The Dental Protective Association. - - - - - 44

Invitation to the Tenth International Medical Congress. - 46

MONTHLY SUMMARY.

Mouth-Breathing and the Teeth. - - - - - 47

A Case in Practice. - - - - - . - 48

☞ The American Journal of Dental Science will contain fifty pages of reading matter in each number, making a volume of about Six Hundred pages,

EXCLUSIVE OF ADVERTISEMENTS.

